# Sirt1 improves heart failure through modulating the NF-κB p65/microRNA-155/BNDF signaling cascade

**DOI:** 10.18632/aging.103640

**Published:** 2020-11-18

**Authors:** Bin Lin, Hui Zhao, Li Li, Zhenzhen Zhang, Nan Jiang, Xiaowei Yang, Tao Zhang, Bowen Lian, Yaokai Liu, Chi Zhang, Jiaxiang Wang, Feng Wang, Deguang Feng, Jing Xu

**Affiliations:** 1 Department of Cardiovascular Surgery, The First Affiliated Hospital of Zhengzhou University, Zhengzhou 450052, P.R. China

**Keywords:** heart failure, Sirt1, NF-κB p65, microRNA-155, BDNF

## Abstract

Heart failure (HF) affects over 26 million people worldwide, yet the pathologies of this complex syndrome have not been completely understood. Here, we investigated the involvement of deacetylase Sirtuin 1 (Sirt1) in HF and its downstream signaling pathways. A HF model was induced by the ligation of the left coronary artery in rats, where factors associated with left ventricular echocardiography, heart hemodynamics and ventricular mass indexes were recorded. Collagen volume fraction in heart tissues was determined by Masson’s trichrome staining. Cell models of HF were also established (H_2_O_2_, 30 min) in cardiomyocytes harvested from suckling rats. HF rats presented with downregulated expressions of Sirt1, brain-derived neurotrophic factor (BDNF) and exhibited upregulated expressions of NF-κB p65 and miR-155. Repressed Sirt1 expression increased acetylation of NF-κB p65, resulting in the elevation of NF-κB p65 expression. NF-κB p65 silencing improved heart functions, decreased ventricular mass and reduced apoptosis in cardiomyocytes. MiR-155 inhibition upregulated its target gene BDNF, thereby reducing cardiomyocyte apoptosis. Sirt1 overexpression upregulated BDNF, improved heart function, and reduced apoptosis in cardiomyocytes. In conclusion, Sirt1 alleviates HF in rats through the NF-κB p65/miR-155/BDNF signaling cascade.

## INTRODUCTION

Heart failure (HF) is a global health burden, affecting more than 26 million people [1]. It is a complex clinical syndrome that may be caused by functional and/or structural impairment of the heart [2]. Although HF can be caused by different etiologies, there is a growing patient population that have HF, with left ventricular impairment and ejection deficiency, which currently does not have any specific treatment guidelines [3]. Half of the HF patients were hypertensive in China; however, approximately 30% were using anti-hypertensive medications [4]. The average duration of hospitalization of HF patients in China was 9-10 days, thereby exerting a huge economic burden on society [5]. Cardiomyocyte apoptosis has been reported as one of the causes for adverse remodeling, thus contributing to HF at later phase [6]. It is, therefore, of great importance to inhibit cardiomyocyte apoptosis to improve the treatment and outcomes of HF.

Ineffective treatment for HF is partially attributed to complex pathologies that have not been fully understood. Sirtuin (Sirt) is a family of highly conserved histone/protein deacetylases that may be important for the treatment of HF [7]. Among Sirt family, Sirt1 and Sirt3 have been extensively studied in the cardiovascular system. Sirt1 is particularly noteworthy because of its effect on cardiomyocyte survival and growth under stress, which is associated with ventricular hypertrophy [8]. Moreover, Sirt1 protects cardiomyocytes against oxidative stress, ischemia/reperfusion injury, and apoptosis [9]. Sirt1 has an inhibitory effect on nuclear factor kappa-light-chain-enhancer of activated B cells (NF-κB) through its deacetylation properties [10, 11], which is an important transcription factor for the immune response, production of pro-inflammatory cytokines, and cell survival [12]. Sirt1 expression has been found to be reduced in HF that may be related to increased levels of acetylation found in HF [13]. Based on the above findings, one would expect that decreased Sirt1 leads to an increased expression of NF-κB in HF. Therefore, our aim was to determine if Sirt1 would be protective against HF through inhibiting the NF-κB p65 subunit, which is related to Sirt1 [14, 15].

NF-κB p65 is highly expressed in HF and promotes microRNA-155 (miR-155) expression [16–18], which promotes HF [19]. MiR-155 is recently found to be a regulator of brain-derived neurotrophic factor (BNDF), with the expression of BNDF being reduced in HF [20–23]. These previous findings led us to further determine the downstream molecular mechanism of NF-κB p65-mediated injury in cardiomyocytes that involves miR-155 and BDNF in HF.

## RESULTS

### Characterization of HF rat models

**Figure 1:**
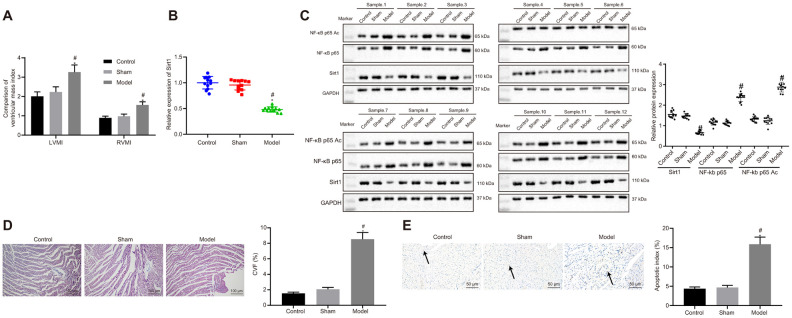
**Sirt1, NF-κB p65 expression and apoptosis in heart tissues of successfully induced HF rats.** (**A**) Ventricular mass index; (**B**) Sirt1 mRNA expression, determined using RT-qPCR; (**C**) Sirt1, NF-κB p65, and NF-κB p65 Ac protein expression assessed by Western blot analysis; (**D**) Collagen volume faction determined by Masson’s trichrome staining (100 ×); (**E**) Apoptosis determined by TUNEL staining (200 ×); *p < 0.05 vs. control rats and # *p* < 0.05 vs. sham rats. Data were expressed as a mean ± standard deviation. Three or more groups were analyzed by one-way analysis of variance (ANOVA) and Tukey's post hoc test. N = 12.

We initially established an animal rat model with HF after myocardial infarction according to the method of left coronary artery ligation. Ultrasonic electrocardiogram test results suggested that HF rats had higher interventricular septal dimension (IVSD), left ventricular end diastolic diameter (LVEDD), left ventricular end systolic diameter (LVESD). In contrast, HF rats had decreased left ventricular posterior wall thickness (LVPWD), left ventricular ejection fraction (LVEF), and fractional shortening (FS) compared to that of control or sham rats (Table 1). The LVEF in HF rats were less than or equal to 45%. There were no differences in LVEF between the control and sham rats. Hemodynamic measurements revealed that left ventricular end diastolic pressure (LVEDP) was significantly elevated, while left ventricular systolic pressure (LVSP), left ventricular pressure (+dp/dt) and the rate of decrease in left ventricular pressure (-dp/dt) were significantly decreased in HF rats compared to that of control and sham rats (Table 2). Both left ventricular mass index (LVMI) and right ventricular mass index (RVMI) were significantly higher in HF rats than those of control and sham rats (Figure 1A).

**Table 1:** Echocardiographic data in all groups of rats.

**Group**	**LVPWD (mm)**	**IVSD (mm)**	**LVEDD (mm)**	**LVESD (mm)**	**LVEF (%)**	**FS (%)**
Control	1.58 ± 0.17	1.44 ± 0.15	4.01 ± 0.42	2.08 ± 0.24	71.14 ± 8.07	25.16 ± 3.34
Sham	1.67 ± 0.17	1.51 ± 0.21	4.17 ± 0.43	2.26 ± 0.31	73.41 ± 7.56	27.15 ± 3.64
HF	0.79 ± 0.08*	2.84 ± 0.35*	7.46 ± 0.84*	4.29 ± 0.51*	34.16 ± 3.62*	16.34 ± 2.04*

Note: *p < 0.05 vs. control and sham rats. HF, heart failure; LVPWD, left ventricular posterior wall thickness; IVSD, interventricular septal dimension; LVEDD, left ventricular end diastolic diameter; LVEF, left ventricular ejection fraction; FS, fractional shortening.

**Table 2:** Cardiac hemodynamic parameters in all groups of rats.

**Group**	**LVSP (mm Hg)**	**LVEDP (mm Hg)**	**HR (bpm)**	**+dp/dt (mm Hg/s)**	**-dp/dt (mm Hg/s)**
Control	115.41±15.16	6.16±0.63	294.45±30.01	4497.45±340.56	2768.57±312.08
Sham	112.15±10.24	7.75±0.82	276.81±35.07	4167.61±486.43	2506.34±401.07
HF	57.16±6.01*	24.67±2.64*	265.16±26.79	1976.37±204.16*	1046.31±186.19*

Note: *p < 0.05 vs. control rats. # *p* < 0.05 vs. sham rats. LVSP, left ventricular systolic pressure; LVEDP, left ventricular end diastolic pressure; HR, heart rate; +dp/dt, rate of increase in left ventricular pressure; -dp/dt, rate of decrease in left ventricular pressure.

### Sirt1 is poorly expressed while NF-κB p65 is highly expressed in heart tissues rats with HF

Messenger RNA (Figure 1B) and protein (Figure 1C) expressions of Sirt1 were determined using reverse transcription quantitative polymerase chain reaction (RT-qPCR) and western blot analysis. The results displayed significantly reduced mRNA and protein Sirt1 expressions in heart tissues of HF rates in contrast with that of control or sham rats. On the other hand, protein expression of NF-κB p65 and NF-κB p65 Ac was significantly increased in HF rats when compared to control and sham rats (Figure 1C). Collagen volume fraction (CVF) (Figure 1D) and cardiomyocytes apoptosis (Figure 1E) were increased in HF rats compared to that of control or sham rats. The above results indicated that Sirt1 expression was elevated, NF-κB p65 in heart tissues of HF rats was upregulated, while acetylation of NF-κB p65 was inhibited in HF rats.

### NF-κB p65 silencing alleviates HF

**Figure 2:**
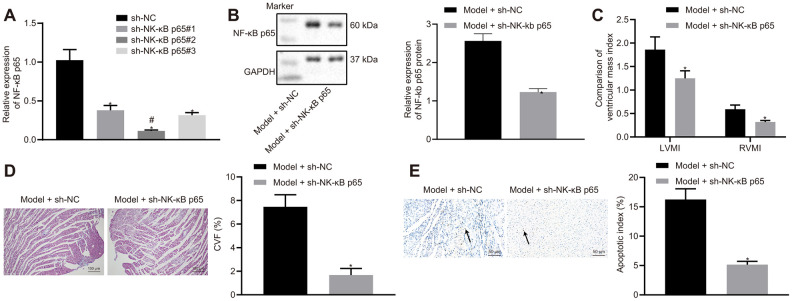
**Effects of NF-κB p65 silencing on heart failure in rats.** (**A**) NF-κB p65 mRNA expression after sh-RNA treatment; (**B**) NF-κB p65 protein expression in heart tissues; (**C**) Ventricular mass index; (**D**) CVF determined by Masson’s trichrome staining (100 ×); (**E**) Apoptosis determined by TUNEL staining (200 ×); *p < 0.05 vs. model + sh-NC group; # indicates lowest NF-κB p65 mRNA expression. Data are expressed as mean ± standard deviation. A comparison of data from 2 groups was performed by an unpaired t test, while 3 or more groups were performed by one-way analysis of variance (ANOVA) and Tukey's post hoc test. N= 12.

Since NF-κB p65 expression was increased in HF rats, we hypothesized that the silencing of NF-κB p65 would improve the outcome of HF in rats. In order to test this hypothesis, we silenced NF-κB p65 expression. Among 3 sh-RNA, sh-NF-κB p65#2 reduced NF-κB p65 most significantly and therefore was chosen for subsequent experiments (Figure 2A). sh-NF-κB p65 also reduced the protein expression of NF-κB p65 when compared to its NC (Figure 2B). NF-κB p65 silencing reduced IVSD, LVEDD and LVESD, while increasing LVPWD, LVEF, and FS in HF rats (Table 3). NF-κB p65 silencing also reduced LVEDP and increased LVSP, +dp/dt, and -dp/dt in hemodynamic measurements. However, the heart rate (HR) of rats among different groups did not vary noticeably (Table 4). NF-κB p65 silencing also reduced LVMI and RVMI (Figure 2C), CVF (Figure 2D), and cardiomyocytes apoptosis (Figure 2E) in HF rats. Collectively, NF-κB p65 silencing alleviated HF.

**Table 3:** Effects of NF-κB p65 silencing on heart functions.

**Group**	**LVPWD (mm)**	**IVSD (mm)**	**LVEDD (mm)**	**LVESD (mm)**	**LVEF (%)**	**FS (%)**
Model + sh-NC	1.07±0.12	2.64±0.31	6.17±0.72	4.12±0.43	32.76±4.54	15.23±1.74
Model + sh-NF-κB p65	1.36±0.19*	1.24±0.16*	2.91±0.39*	1.54±0.21*	67.34±7.84*	23.16±2.69*

Note: *p < 0.05 vs. model + sh-NC group. NC, negative control; miR-155, microRNA-155; BNDF, brain-derived neurotrophic factor; LVPWD, left ventricular posterior wall thickness; IVSD, interventricular septal dimension; LVEDD, left ventricular end diastolic diameter; LVEF, left ventricular ejection fraction; FS, fractional shortening.

**Table 4:** Effects of NF-κB p65 silencing on hemodynamics of the heart.

**Group**	**LVSP (mm Hg)**	**LVEDP (mm Hg)**	**HR (bpm)**	**+dp/dt (mm Hg/s)**	**-dp/dt (mm Hg/s)**
Model + sh-NC	48.26 ± 4.91	20.13 ± 2.46	276.15 ± 31.24	1724.71 ± 186.72	996.14 ± 100.26
Model + sh- NF-κB p65	104.37±11.28*	5.01±0.72*	281.47±33.24	4067.38±412.34*	1817.21±204.37*

Note: *p < 0.05 vs. model + sh-NC group. NC, negative control; LVSP, left ventricular systolic pressure; LVEDP, left ventricular end diastolic pressure; HR, heart rate; +dp/dt, rate of increase in left ventricular pressure; -dp/dt, rate of decrease in left ventricular pressure.

### NF-κB p65 binds to the promoter region and elevates miR-155 expression in cardiomyocytes

**Figure 3:**
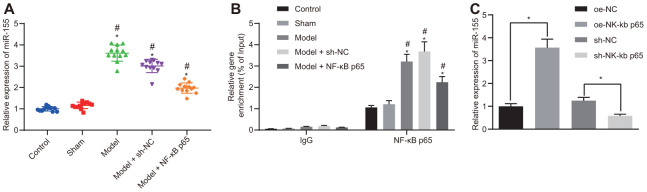
**Binding relationship between NF-κB p65 and miR-155.** (**A**) miR-155 expression in heart tissues; (**B**) Enrichment of NF-κB p65 in the promoter region of miR-155 determined by ChIP assay; (**C**) miR-155 expression in cardiomyocytes; *p < 0.05 vs. control, oe-NC or sh-NC group; #*p* < 0.05 vs. sham group; ^*p* < 0.05 vs. model + sh-NC group. Data were expressed as a mean ± standard deviation. Three or more groups by one-way analysis of variance (ANOVA) and Tukey's post hoc test. N= 12.

The expression of miR-155 was significantly higher in HF rats and was partially reduced after NF-κB p65 silencing (Figure 3A). MiR-155 expression remained unchanged in the control and sham rats. The enrichment of NF-κB p65 in the miR-155 promoter region was significantly higher in HF rat cardiomyocytes, which was subsequently reduced by NF-κB p65 silencing (Figure 3B). Furthermore, NF-κB p65 overexpression increased miR-155 expression, whereas NF-κB p65 silencing reduced miR-155 expression (Figure 3C). These results showed that NF-κB p65 upregulated miR-155 by binding to the promoter region of miR-155.

### MiR-155 inhibition reduces cardiomyocyte apoptosis in cell models of HF

**Figure 4:**
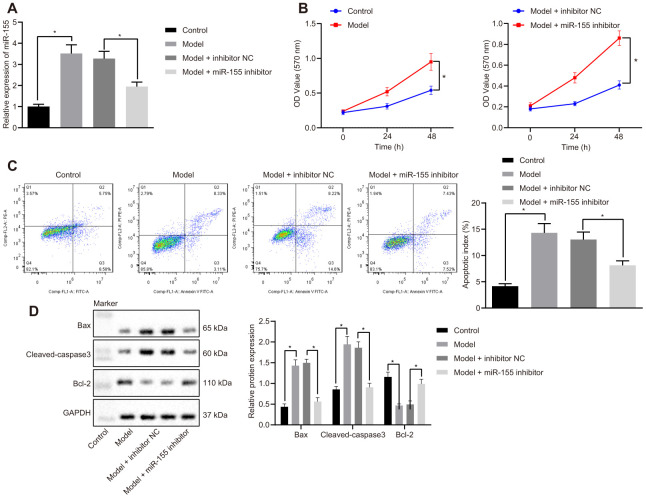
**Effects of miR-155 inhibition on cardiomyocyte apoptosis.** (**A**) Expression of miR-155; (**B**) Cell viability determined by MTT assay; (**C**) Cell apoptosis determined by flow cytometry; (**D**) Protein expression of apoptosis-related factors; *p < 0.05 vs. control group or model + inhibitor NC. Data were expressed as a mean ± standard deviation. Three or more groups were analyzed by one-way analysis of variance (ANOVA) and Tukey's post hoc test. Data comparison between different time points was performed by repeated measures ANOVA and Bonferroni post hoc test. The cell experiment was repeated three times.

MiR-155 expression was increased in cardiomyocytes treated with H_2_O_2_ and reduced by a miR-155 inhibitor (Figure 4A). Cell viability in HF cells was significantly lower than that of the control cells (left panel, Figure 4B). The miR-155 inhibitor significantly increased cell viability in HF cells (right panel, Figure 4B). H_2_O_2_ treatment also increased apoptosis and was normalized by an miR-155 inhibitor (Figure 4C). Similarly, H_2_O_2_ treatment increased apoptosis-related proteins Bax and cleaved-caspase3 prominently while at the same time decreased anti-apoptosis-related Bcl-2 protein (Figure 4D). The miR-155 inhibitor reversed the aforementioned effects mentioned above. These results suggested that miR-155 reduced cardiomyocyte apoptosis in HF.

### MiR-155 downregulates BDNF and promotes cardiomyocyte apoptosis

**Figure 5:**
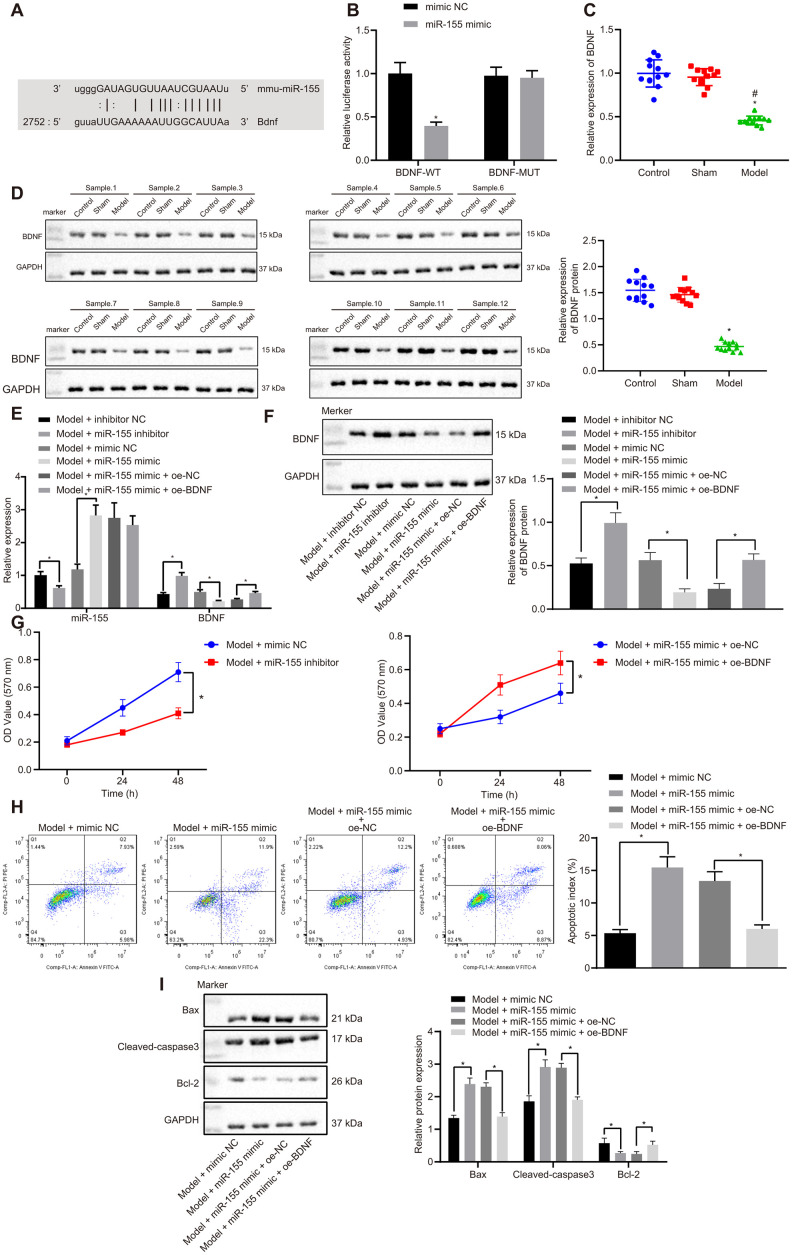
**Effects of miR-155 and BDNF on cardiomyocyte apoptosis.** (**A**) Binding relationship between miR-155 and BDNF predicted by online tools; (**B**) Binding relationship between miR-155 and BDNF determined by dual luciferase reporter gene assay; (**C**) BDNF mRNA expression in heart tissues; (**D**) BDNF protein expression in heart tissues; (**E**) miR-155 and BDNF mRNA expression in cardiomyocytes. (**F**) BDNF protein expression in cardiomyocytes; (**G**) Cell viability determined by MTT assay; (**H**) Cell apoptosis determined by flow cytometry; (**I**) Protein expression of apoptosis-related factors; *p < 0.05 vs. control, model + inhibitor NC, model + mimic NC, or model + miR-155 mimic + oe-NC groups; #*p* < 0.05 vs. sham group. Data were expressed as a mean ± standard deviation. A comparison of data from 2 groups was performed by an unpaired t test, while 3 or more groups by one-way analysis of variance (ANOVA) and Tukey's post hoc test. Data comparison between different time points was performed by repeated measures ANOVA and Bonferroni post hoc test. N= 12. The cell experiment was repeated three times.

Online database mirDIP, RNA22, miRWalk, and starBase searches predicted that BDNF has a binding site for miR-155 (Figure 5A). We further studied this binding relationship by using a dual luciferase reporter gene assay. Luciferase activity in the BDNF-wild-type (WT)/miR-155 mimic group was significantly lower than that of mimic NC group (Figure 5B). However, there were no differences in luciferase activity between the BDNF-mutated (MUT)/miR-155 mimic group and mimic NC group. This indicates that miR-155 specifically binds to the BDNF (Figure 5B). BDNF mRNA (Figure 5C) and protein (Figure 5D) expressions in the heart of HF rats were significantly lower than that in control and sham rats. These results suggested that BDNF may be related to HF involving miR-155.

In cardiomyocytes treated with H_2_O_2_, the miR-155 inhibitor significantly reduced the mRNA (Figure 5E) and protein (Figure 5F) expressions of miR-155 but on the other hand, increased BDNF levels. Conversely, miR-155 mimic increased miR-155 expression but reduced BDNF. MiR-155 mimic decreased cell viability (Figure 5G) and increased apoptosis (Figure 5H) in HF cardiomyocytes. BDNF overexpression normalized the effect of miR-155 mimic. Similarly, miR-155 mimic was found to upregulate Bax and cleaved-caspase3 while decreasing Bcl-2, but this trend was prevented by BDNF overexpression (Figure 5I). These results indicated miR-155 overexpression inhibited BDNF expression and promoted cardiomyocyte apoptosis. Therefore, miR-155 inhibition up-regulated BDNF and reduced cardiomyocytes apoptosis and thus may be beneficial to HF.

### MiR-155/BDNF axis attenuates HF *in vivo*

**Figure 6:**
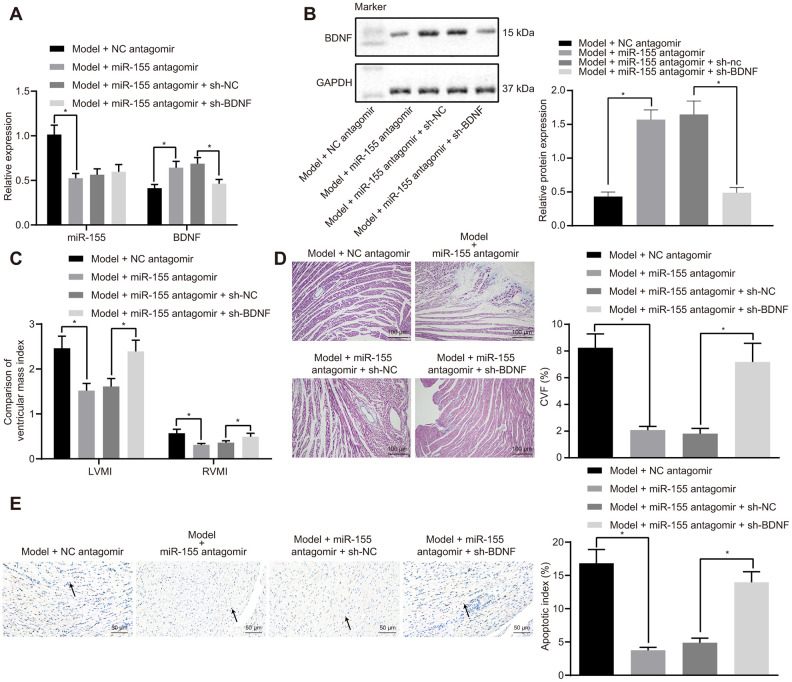
**Effects of miR-155 and BDNF on heart failure *in vivo*.** (**A**) Expression of miR-155 and BDNF mRNA expression; (**B**) BDNF protein expression; (**C**) Ventricular mass index; (**D**) CVF determined by Masson’s trichrome staining (100 ×); (**E**) Apoptosis determined by TUNEL staining (200 ×); *p < 0.05 vs. model + NC antagomir or model + miR-155 antagomir + sh-NC group. Data were expressed as mean ± standard deviation. A comparison of data from 2 groups were performed by unpaired t test. N= 12.

MiR-155 antagomir significantly decreased mRNA (Figure 6A) and protein (Figure 6B) expressions of miR-155 while increasing that of BDNF in heart tissues of HF rats. The addition of sh-BDNF exhibited no effect on the expression of miR-155, but decreased that of BDNF in miR-155 antagomir-treated HF rats. MiR-155 antagomir decreased IVSD, LVEDD, and LVESD while increasing LVPWD, LVEF, and FS in HF rats (Table 5). BDNF silencing significantly increased IVSD, LVEDD, and LVESD and decreased LVPWD, LVEF, and FS in miR-155 antagomir-treated HF rats. MiR-155 antagomir decreased LVEDP while increasing LVSP, +dp/dt, and -dp/dt (Table 6). As expected, BDNF silencing significantly increased LVEDP and decreased LVSP, +dp/dt, and -dp/dt in miR-155 antagomir-treated HF rats. There was no noteworthy difference in HR between groups. LVMI, RVMI (Figure 6C), CVF (Figure 6D), and cardiomyocytes apoptosis (Figure 6E) were significantly lowered by miR-155 antagomir and were normalized by BDNF silencing in heart tissues of HF rats. These results indicated that miR-155 inhibition upregulated BDNF expression and alleviated HF *in vivo*.

**Table 5:** Effects of miR-155 and BDNF inhibition on heart functions in HF rats.

**Group**	**LVPWD (mm)**	**IVSD (mm)**	**LVEDD (mm)**	**LVESD (mm)**	**LVEF (%)**	**FS (%)**
Model + NC antagomir	0.84±0.09	1.37±0.34	3.96±0.54	1.92±0.39	33.17±1.37	11.64±1.27
Model + miR-155 antagomir	1.23±0.16*	0.78±0.17*	1.25±0.34*	1.01±0.21*	68.16±2.76*	23.38±2.14*
Model + miR-155 antagomir + sh-NC	1.34±0.15	0.62±0.14	1.42±0.28	1.16±0.23	69.14±2.72	23.08±2.31
Model + miR-155 antagomir + sh-BDNF	0.76±0.08*	1.41±0.29*	3.73±0.79*	2.07±0.32*	31.05±1.34*	11.25±0.96*

Note: *p < 0.05 vs. model + NC antagomir or model + miR-155 antagomir + sh-NC group. HF, heart failure; NC, negative control; miR-155, microRNA-155; BNDF, brain-derived neurotrophic factor; LVPWD, left ventricular posterior wall thickness; IVSD, interventricular septal dimension; LVEDD, left ventricular end diastolic diameter; LVEF, left ventricular ejection fraction; FS, fractional shortening.

**Table 6:** Effects of miR-155 and BDNF inhibition on heart hemodynamics in HF rats.

**Group**	**LVSP (mm Hg)**	**LVEDP (mm Hg)**	**HR (bpm)**	**+dp/dt (mm Hg/s)**	**-dp/dt (mm Hg/s)**
Model + NC antagomir	53.64±5.86	22.46±2.47	249.21±26.85	1921.26±203.16	1017.24±129.24
Model + miR-155 antagomir	93.34±9.46*	5.34±0.71*	261.37±25.89	4054.23±463.79*	2364.69±217.45*
Model + miR-155 antagomir + sh-NC	109.56±15.24	4.21±0.63	279.34±31.26	4212.34±465.79	2169.37±301.75
Model + miR-155 antagomir + sh-BDNFl	49.67±5.79*	21.04±2.76*	278.76±34.19	1863.27±197.64*	996.17±107.65*

Note: *p < 0.05 vs. model + NC antagomir or model + miR-155 antagomir + sh-NC group. HF, heart failure; NC, negative control; miR-155, microRNA-155; BNDF, brain-derived neurotrophic factor; LVSP, left ventricular systolic pressure; LVEDP, left ventricular end diastolic pressure; HR, heart rate; +dp/dt, rate of increase in left ventricular pressure; -dp/dt, rate of decrease in left ventricular pressure.

### Sirt1 overexpression alleviates HF *in vivo*

**Figure 7:**
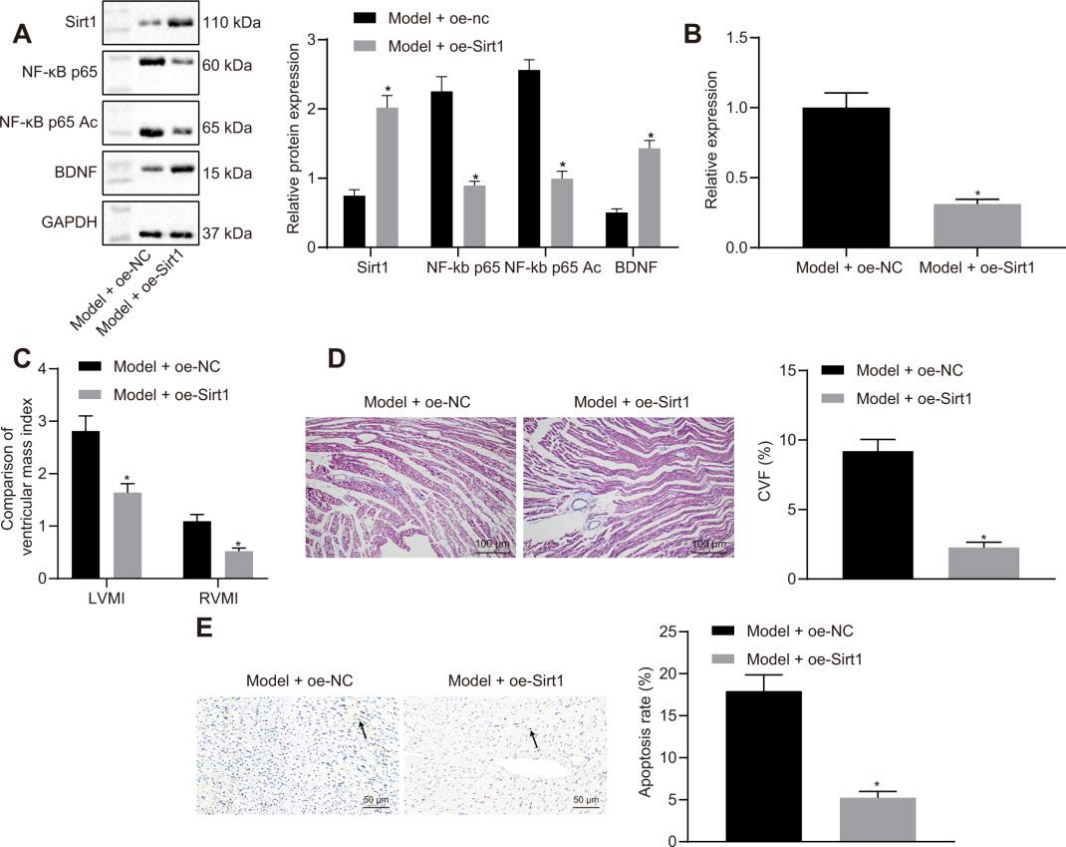
**Effects of Sirt1 overexpression on heart failure *in vivo*.** (**A**) Sirt1, NF-κB p65, NF-κB p65 Ac and BDNF protein expression; (**B**) miR-155 expression; (**C**) Ventricular mass index; (**D**) CVF determined by Masson’s trichrome staining (100 ×); (**E**) Apoptosis determined by TUNEL assay (200 ×); *p < 0.05 vs. model + oe-NC group. Data were expressed as mean ± standard deviation. A comparison of data from 2 groups was performed by an unpaired t test. N= 12.

Sirt1 overexpression reduced NF-κB p65 and NF-κB p65 Ac protein expressions in heart tissues of HF rats (Figure 7A). Sirt1 overexpression led to increased BDNF expression. Moreover, Sirt1 overexpression reduced miR-155 expression (Figure 7B). These results indicated that the overexpression of Sirt1 downregulated NF-κB p65 by inhibiting NF-κB p65 acetylation, thus leading to reduced miR-155 expression and an increased BDNF expression.

Sirt1 overexpression reduced IVSD, LVEDD, and LVESD while elevated LVPWD, LVEF, and FS in HF rats (Table 7). Heart hemodynamic studies showed that LVEDP was significantly decreased, while LVSP, +dp/dt, and -dp/dt were increased by Sirt1 overexpression (Table 8). The HR was not different among the different groups. LVMI, RVMI (Figure 7C), CVF (Figure 7D), and cardiomyocytes apoptosis (Figure 7E) were reduced in HF rats by the overexpression of Sirt1. These results indicated that the overexpression of Sirt1 exhibited protective effects against HF.

**Table 7:** Effects of Sirt1 overexpression on heart functions in HF rats.

**Group**	**LVPWD (mm)**	**IVSD (mm)**	**LVEDD (mm)**	**LVESD (mm)**	**LVEF (%)**	**FS (%)**
Model + oe-NC	0.97±0.12	1.36±0.14	3.65±0.84	1.87±0.24	34.18±3.76	17.08±1.19
Model + oe-Sirt1	1.42±0.16*	0.68±0.12*	1.62±0.23*	0.97±0.15*	70.31±7.79*	24.07±3.06*

Note: *p < 0.05 vs. model + oe-NC group. HF, heart failure; NC, negative control; LVPWD, left ventricular posterior wall thickness; IVSD, interventricular septal dimension; LVEDD, left ventricular end diastolic diameter; LVEF, left ventricular ejection fraction; FS, fractional shortening.

**Table 8:** Effects of Sirt1 overexpression on heart hemodynamics in HF rats.

**Group**	**LVSP (mm Hg)**	**LVEDP (mm Hg)**	**HR (bpm)**	**+dp/dt (mm Hg/s)**	**-dp/dt (mm Hg/s)**
Model + oe-NC	58.49±6.94	25.46±2.75	272.45±32.16	2106.28±234.67	1123.71±167.49-
Model + oe-Sirt1	105.34±12.46*	5.24±0.61*	251.64±34.16	4316.94±310.37*	2696.49±302.57*

Note: *p < 0.05 vs. model + oe-NC group. HF, heart failure; NC, negative control; LVSP, left ventricular systolic pressure; LVEDP, left ventricular end diastolic pressure; HR, heart rate; +dp/dt, rate of increase in left ventricular pressure; -dp/dt, rate of decrease in left ventricular pressure.

## DISCUSSION

HF is a complex disease that has a wide variety of etiological causes and mechanisms of action, many of which are still not properly understood. There are multiple animal and cell models to mimic certain, but not all, aspects of HF [24, 25]. In this study, we used an artery ligation rat model to mimic ischemic injury and an H_2_O_2_-induced cardiomyocyte injury cell model. Collectively, reduced Sirt1 expression in HF lead to increased NF-κB p65 and miR-155 expressions, reduced BDNF expression, which promoted HF.

Sirt1 expression is shown to be reduced in HF [13]. The reduced expression of Sirt1 in HF is consistent to the increased acetylation in HF [26, 27]. Similarly, we found that a reduction in Sirt1 expression produced an increase in levels of acetylation and expression of NF-κB p65. Therefore, we expected that increased Sirt1 expressions would prevent injuries in cardiomyocytes and hence avoid the subsequent progression to HF. We also discovered that Sirt1 overexpression increased cell viability, reduced apoptosis, and improved heart function in HF rats. These results agree with previous studies by showing the beneficial effects of Sirt1 on HF including: improving cell survival, reducing oxidative stress, and reducing ischemia/reperfusion injury [8, 9, 28, 29]. Therefore, Sirt1 may be a novel target for the treatment of HF [7, 30].

The increased levels of protein acetylation are well-known in HF. It is postulated that acetylation is caused by ischemic stress, leading to metabolic dysfunction especially in mitochondria [26]. The acetylation of metabolic-relevant, non-histone-related proteins such as ATP synthase, long-chain acyl-CoA dehydrogenase, creatine kinase, malate dehydrogenase, and pyruvate dehydrogenase can be visibly seen in the mitochondria of failing hearts [31]. Acetylation of histone-related proteins, on the other hand, has been shown to be responsible for an increased heart mass, such as left ventricular hypertrophy in HF [32]. In this study, we reported to the best of our knowledge, that the acetylation of NF-κB was increased in heart tissues during HF because of reduced deacetylase Sirt1. Our results supported the idea that acetylation is a potential target for treating HF [33, 34].

In the subsequent experiments, we found that a reduced Sirt1 expression led to an increased level of NF-κB. This result was in line with previous findings that Sirt1 exerts an inhibitory effect on NF-κB [10, 11]. NF-κB is a transcription factor that involves many biological and pathological functions. It is speculated that NF-κB may cause overactive immune and/or inflammatory responses that promotes injury in HF [35]. On the other hand, NF-κB may promote HF by directly initiating cell death in cardiomyocytes [36]. The effects of NF-κB on HF may involve its target gene, miR-155, which is shown in previous studies [16, 17]. MiR-155 may be involved in HF based on a few observations. Our results showed that miR-155 inhibition decreased ventricular hypertrophy, cardiomyocyte apoptosis and increased cardiac functions. Moreover, a previous study demonstrated that miR-155 is highly expressed in HF and has been proposed to promote ventricular hypertrophy and dysfunction [17, 19]. Our results also presented that miR-155 overexpression increased cardiomyocyte apoptosis and expression of BDNF. Consistently, the inhibition of miR-155 suppresses the cardiomyocyte apoptosis induced by myocardial ischemia/reperfusion [37] or endoplasmic reticulum stress [38].

MiR-155 has been recently found to regulate BNDF [20, 21]. Similarly in our study, we found that miR-155 mimic reduced BDNF expression. Although the majority of the research on BNDF has been conducted on the brain, one report elucidates the role of BNDF in the neuronal-cardiac link [39]. For example, BNDF may have a role in the neuronal control of contraction and relaxation in cardiomyocytes, therefore having a physiological role in regulating heartbeat [40]. BNDF may also be protective against ischemia/reperfusion-induced cell deaths in the heart [41]. Our results were similar in a way that BNDF silencing prevented many beneficial effects of miR-155 inhibition including: reduced cardiomyocytes apoptosis, ventricular hypertrophy, and improved heart function *in vivo*. These results collectively provide strong evidence that reduced BNDF may have a role in causing ventricular dysfunction in HF. In other words, increased BNDF expression in cardiomyocytes may be beneficial to heart function in HF.

## CONCLUSIONS

In conclusion, this study suggests that increased NF-κB expression in cardiomyocytes, induced by the attenuated expression of Sirt1, upregulates miR-155. An increased expression of miR-155 in cardiomyocytes inhibits the expression of BNDF, leading to ventricular dysfunction in HF. All signaling molecules in this pathway warrant further investigation as potential pharmaceutical targets against injuries in cardiomyocytes during HF. Additionally, other models should also be explored and help confirm results from this study because these two models only mimic part of the human disease.

## MATERIALS AND METHODS

All animal procedures were carried out in accordance with the Guide for the Care and Use of Laboratory Animals published by the National Institutes of Health and approved by the Animal Ethics Committee of The First Affiliated Hospital of Zhengzhou University.

### Establishment of a rat model of HF

Healthy male Sprague-Dawley rats (207 ± 20 g, n = 120, Animal Experimental Center, Guangzhou University of Traditional Chinese Medicine, Guangzhou, Guangdong, China) were kept in a pathogen-free animal facility at (22 ± 3)°C, with a relative humidity 40-70%, in a 12 hour/12 hour light/dark cycle with free access to food and water. Rats were randomly divided into control (n = 12), sham (n = 12), and HF groups (n = 96). HF in rats was induced by the ligation of the left coronary artery between the left atrial appendage and the pulmonary artery, as described previously [42]. Sham rats received coronary artery exposure and suture threaded without ligation. Four weeks after the surgery, echocardiographic determination (SonoAce X8, Jumu Medical Devices Co., Ltd., Shanghai, China) of LVEF ≤ 45% was used to make sure that HF was induced successfully [43, 44]. Animals with unsuccessful induction of HF were replaced.

### Transfection in HF rats

HF rats were subdivided into 8 groups (n = 12 per group): (1) model + sh-negative control (NC) (NK-κB p65 silencing negative sequence); (2) model + sh-NF-κB p65 (NF-κB p65 silencing plasmid); (3) model + NC antagomir (miR-155 antagomir negative sequence); (4) model + miR-155 antagomir (miR-155 antagomir oligonucleotide); (5) model + miR-155 antagomir + sh-NC (miR-155 antagomir oligonucleotide + BDNF silencing negative sequence); (6) model + miR-155 antagomir + sh-BDNF (miR-155 antagomir oligonucleotide + BDNF silencing plasmid); (7) model + oe-NC (Sirt1 overexpression negative sequence); and (8) model + oe-Sirt1 (Sirt1 overexpression plasmid). A transfecting solution (0.5 nM, in 3 μL phosphate buffered saline, PBS) was injected through the tail vein according to instructions provided by the En-transter^TM^-*in vivo* kit [45]. The control group received 3 μL of PBS [46]. All sequencing and packaging of viral vectors were prepared by GeneChem (Shanghai, China).

### Left ventricular echocardiography in HF rats

Rats were anesthetized by pentobarbital sodium (3%, i.p., P3761, Sigma, St. Louis, MO, USA) and secured on a wooden board in the supine position. Hair near the chest area was removed for Doppler ultrasonography (SSI-5000, ShuKang HengTong Science and Trade, Shandong, China) to determine the following: LVPWD, IVSD, LVEDD, LVESD, LVEF and FS.

### Heart hemodynamics in HR rats

Rats were anesthetized with pentobarbital sodium and secured on an operating table in the supine position. LVSP, LVEDP, HR, +dp/dt and -dp/dt were simultaneously recorded on a multi-channel physiological recorder (p3 plus, B&E Teksystems, Beijing, China).

### Ventricular mass index in HF rats

Rats’ hearts were harvested and placed in a pre-cooled hydroxyethyl piperazine ethanesulfonic acid (HEPES) solution. The right and left ventricles were separated and placed on an electronic balance for determining the right ventricular mass and left ventricular mass. LVMI and RVMI were calculated by ventricular mass/body weight. Heart tissues were then fixed in 10% formaldehyde (pH 7.0) for 24 hours, embedded in paraffin, and cut into 4 μm coronal sections. Histopathological changes were observed under a microscope.

### RT-qPCR

The tissue or cell homogenate (100 μL) was completely mixed with 1 mL Trizol reagent (15596-018, SolarBio Life Sciences, Beijing, China). Chloroform (200 μL) was added, mixed, and allowed to sit at room temperature for 15 min. The mixture was then centrifuged at 12,000 rpm for 15 min at 4°C. Supernatant was obtained, mixed with 0.5 mL of isopropanol, and allowed to sit for 10-30 min at room temperature. The resulting mixture was centrifuged at 12,000 rpm for 10 min at 4°C. Supernatant was discarded and RNA was precipitated. The RNA was resuspended with 1 mL of 75% ethanol and diluted with 20 μL of diethyl pyrocarbonate (DEPC)-treated water. The mixture was centrifuged at 8,000 rpm for 5 min at 4°C. Supernatant was discarded and the pellet was allowed to dry at room temperature for 5-10 min. The pellet was resuspended in 20 μL of DEPC-treated water to have its RNA concentration determined. RNA (2 μg) was used to produce cDNA by TaqMan reverse transcription reagent (Roche, Basel, Switzerland). Target genes were amplified by PCR in a 50 μL reaction system. Primer (Sigma, St. Louis, MO, USA) sequences are presented in Table 9. All samples were tested in triplicates. Glyceraldehyde phosphate dehydrogenase (GAPDH) was used as an internal reference primer for Sirt1, NF-κB p65, and BNDF. U6 was used as an internal reference primer for miR-155. The relative expression of genes to be tested was calculated by using the 2^-ΔΔCT^ method [47].

**Table 9:** Sequence of primers used in RT-qPCR.

**Primer**	**Primer sequence (5'-3')**	
GAPDH	F: CTGACCATGCCGCCTGGAGA	R: ATGTAGGCCATGAGGTCCAC
U6	F: ATGACGTCTGCCTTGGAGAAC	R: TCAGTGTGCTACGGAGTTCAG
Sirt1	F: GTCTGTGCCTTCCAGTTGCT	R: CTGCTTGCTGTCCATACCTG
NF-κBp65	F: GTGCAGAAAGAAGACATTGAGGTG	R: AGGCTAGGGTCAGCGTATGG
MiR-155	F: GGAGGTTAATGCTAATTGTGATAG	R: GTGCAGGGYCCGAGG
BNDF	F: CTGCTTCAGTTGGCCTTTCG	R: TGCTGTGGTGGTGATTGCCTCTGTG

Note: F, forward; R, reverse; GAPDH, glyceraldehyde phosphate dehydrogenase; miR-155, microRNA-155; BNDF, brain-derived neurotrophic factor.

### Western blot analysis

Heart tissue homogenate (100 μL) or cell lysate (1 mL) was digested at 4°C for 30 min. The mixture was centrifuged at 12,000 rpm for 20 min at 4°C. The protein concentration of the supernatant was determined by bicinchoninic acid kits (20201ES76, Yeasen Biotechnology, Shanghai, China). Lysate volume was diluted to only allow each sample to contain 30 μg protein. Samples were mixed with a loading buffer and boiled at 100°C for 5 min. Samples were then cooled with ice, centrifuged and loaded on sodium dodecyl sulfate polyacrylamide gel electrophoresis separation gel (10%) for electrophoresis. The proteins were transferred onto a nitrocellulose membrane and blocked by 5% skim milk at 4°C overnight. The membranes were incubated with primary rabbit anti-mouse Sirt1 (1: 2000, ab233398), NF-κB p65 (1: 1000, ab28856), NF-κB Ac (1: 500, ab19870), BDNF (1: 2000, ab108319), caspase-3 (1: 500, ab4051), cleaved-caspase3 (1: 500, ab49822), Bax (1: 2000, ab32503), Bcl-2 (1: 1000, ab59348) and GAPDH (1: 2500, ab9485) antibodies overnight at 4°C on a shaker. The membranes were washed with Tris-buffered saline and Tween 20 (TBST) for 5 min each time for a total of 3 times and were incubated with horseradish peroxidase -labeled secondary goat anti-rabbit IgG (1:5000, ab6721) antibodies for 1 hour at room temperature. The samples were washed by TBST for 5 min each time for a total of 3 times. The membranes were developed and images were taken. Gray intensity in protein bands were analyzed by Quantity One software. GAPDH was used as the internal reference. All samples were tested in triplicates and all antibodies were purchased from Abcam, Cambridge, UK.

### Masson’s trichrome staining

Paraffin-embedded myocardial tissues were dried in an oven at 65°C for 3 hours and dewaxed. Tissues were treated with 10% trichloroacetic acid and 10% potassium dichromate for 40 min each. Tissues were then rinsed with tap water, stained with hematoxylin (PT001, BoGoo Biotechnology Co., Ltd., Shanghai, China) and left alone for 8 min. Tissues were stained with 1% Ponceau S (HL12202, Harling Biotechnology Co., Ltd., Shanghai, China) and 1% Fuchsine (HPBIO-SJ820, Hepeng Biotechnology Co., Ltd., Shanghai, China) for 40 min after being rinsed with tap water. The stain was removed by adding 1% glacial acetic acid, followed by 1% molybdic acid solution. The samples were dehydrated and fixated for observation under a microscope. Basement membrane and collagen fibers were stained blue or green. Immune cells were stained red while nuclei stained blueish brown. Five fields in each sample were randomly selected to be observed under a polarized light microscope. CVF was calculated by Image-Pro plus 5.1 image analysis software (Media Cybernetics, Rockville, MD, USA) based on: CVF (%) = collagen area /full field area × 100%.

### TUNEL staining

Paraffin-embedded heart sections were dewaxed and soaked in 3% H_2_O_2_ methanol solution for 30 min at room temperature. After washing with PBS for 5 min each time for a total of 3 times, proteinase K solution was added to the sections dropwise and incubated at 37°C for 30 min. The sections were incubated in 0.1% Trion X-100 and 0.1% sodium citrate solution for 5 min at room temperature and washed with distilled water for 5 min each time for a total of 3 times. The sections were incubated with a freshly prepared TUNEL reaction mixture (prepared based on kit manufacturer’s instructions) at 37°C for 90 min. The sections were then washed in TBS for 5 min each time for 3 times at room temperature. Bovine serum albumin blocking solution (5%) was added and incubated for 20 min at room temperature in a wet box. POD solution was added and incubated at 37°C for 30 min, followed by washing with TBS for 5 min each time for 2 times. The sections were developed in 3,3′-diaminobenzidine at room temperature under the guidance of a microscope. Color development was stopped by rinsing the sections with water. The cells with brown-yellow granules in the nucleus were defined as TUNEL-positive cells. Five fields were randomly chosen in each sample. The number of TUNEL-positive cells per field, under a 400- or 200-fold microscope, was counted and averaged with a BI-2000 image analysis system. All samples were tested in triplicates.

### HF cell model in primary rat cardiomyocytes from suckling rats

Ventricles from SD rats (2-3-day-old) were obtained and washed with Hank's solution (pH 7.2-7.4) 3 times. Tissues were cut into small pieces and detached with 0.25% trypsin at 37°C for 10 min. Trypsin solution was replaced by fresh 0.25% trypsin and incubated for another 30 min at 37°C. Tissues were washed with Hank's solution again for another 3 times. Cells that were fully dispersed were adjusted to 2 × 10^6^ cells/mL with Dulbecco's modified eagle medium (DMEM) containing 20% fetal bovine serum and were incubated for 4 hours at 37°C in a 5% CO_2_ incubator. Adherent cells were discarded. The survival rate of floating cells was observed after trypan blue staining. When cell survival rate achieved > 95%, cells (100 μL/well) were transferred to a 96-well culture plate and cultured at 37°C with 5% CO_2_ for 7 days. H_2_O_2_ (100 m/L in 10% serum culture medium) was added and incubated for 30 min to induce HF model in cardiomyocytes.

### Transfection in primary rat cardiomyocytes

Cardiomyocytes treated with H_2_O_2_ were divided into 6 transfection groups: (1) model + inhibitor NC (miR-155 inhibitor negative sequence), (2) model + miR-155 inhibitor (miR-155 inhibitor plasmid), (3) model + mimic NC group (miR-155 mimic negative sequence), (4) model + miR-155 mimic (miR-155 mimic plasmid), (5) model + miR-155 mimic + oe-BNDF NC (miR-155 mimic plasmid + BDNF overexpression negative sequence), and (6) model + miR-155 mimic + oe-BDNF (miR-155 mimetic plasmid + BDNF overexpression plasmid). Cardiomyocytes were seeded in a six-well plate for 24 hours before transfection to allow the cell confluence to reach about 70%. Lipofectamine 2000 liposomes (20 μL, 11668019, Thermo Fisher Scientific, Waltham, MA, USA) were diluted in a 500 μL serum-free culture medium and incubated with cardiomyocytes for 5 min at room temperature. Plasmids and liposomes were mixed and incubated for 20 min at room temperature. The cardiomyocytes were washed three times with a serum-free culture medium. The medium was added, incubated with liposome mixture for 5-24 hours, and replaced every 6 hours during transfection. Cardiomyocytes were incubated with 20% antibiotic-free DMEM for 48 hours after being washed three times with serum-free medium. RT-qPCR was used to validate the interference efficiency of shRNA (sh-NF-κB p65#1, sh-NF-κB p65#2, sh-NF-κB p65#3). ShRNA that caused the lowest expression of NF-κB p65 was used for further experiments.

### Chromatin immunoprecipitation (ChIP) assay

The enrichment of NF-κB p65 in the promoter region of miR-155 gene was determined by a ChIP kit (Millipore, Burlington, MA, USA). Cells in the logarithmic growth phase were mixed with 1% formaldehyde for 10 min at room temperature to allow DNA- protein cross-links to form. Cross-links formed were fragmented to an appropriate size by ultrasonic device for 10 seconds for 15 times with 10 second intervals after each cycle. Parts of the cross-link fragments were centrifuged at 13,000 rpm at 4°C, while the remaining fragments were used as ChIP Input. The supernatant was collected into three tubes and incubated with primary rabbit anti-rat NF-κB p65 (ab19870, Abcam, Cambridge, UK) or NC IgG antibodies overnight at 4°C. Endogenous DNA-protein complexes were precipitated by protein agarose/sepharose. After centrifugation, supernatant was discarded. Cross-link proteins were allowed to break down at 65°C overnight. DNA fragments were then purified by phenol/chloroform extraction. The binding of NF-κB p65 to the promoter region of miR-155 (Table 9) was determined using Input as an internal reference.

### Dual luciferase reporter gene assay

WT and MUT site sequences of the 3'-untranslated region (UTR) in BDNF mRNA were digested. Target gene fragments of WT and MUT were inserted into pmiR-RB-REPORT^TM^ vectors (Guangzhou RiboBio Biotechnology Co., Ltd., Guangzhou, China) that had been digested previously with restriction endonucleases. The empty plasmid transfection served as a control. Vectors containing MUT and WT were co-transfected into HEK293T cells with NC mimics or miR-155 mimics, respectively. Cells were collected and lysed, centrifuged for 3-5 min, and allowed the supernatant to be collected after 48 hours of transfection. Relative light units (RLUs) were determined by Renilla Luciferase Assay Kit (YDJ2714, Yuduo Biotechnology Co., Ltd., Shanghai, China) using Firefly luciferase as an internal reference. Results between Renilla and Firefly luciferase were analyzed by using a dual luciferase reporter assay system (Promega Co., Madison, WI, USA).

### 3-(4,5-Dimethyl-2-Thiazyl)-2,5-Diphenyl-2H-Tetrazolium Bromide (MTT) assay

Cardiomyocytes were detached with 0.25% trypsin to prepare single cell suspensions. Cells (0.2 mL) were inoculated at 3-6 × 10^3^ cells/well in 96-well plates (6 wells/group). Each of the culture mediums was replaced by a medium containing 10% MTT solution (5 g/L, GD-Y1317, Gudu Biotech Co., Ltd., Shanghai, China) and allowed to culture for 4 hours at 24-hour, 48-hour and 72-hour time intervals. The supernatant was removed and 100 μL of dimethyl sulfoxide (D5879-100ml, Sigma, St. Louis, MO, USA) was added and fully mixed for 10 min. Formazan crystals produced by living cells were detected by a microplate reader set at 570 nm (BS-1101, Detie Equipment Ltd., Nanjing, China).

### Flow cytometry

Cells were detached with 0.25% trypsin (without ethylene diamine tetraacetic acid, EDTA), centrifuged and the supernatants discarded after transfection for 48 hours. Cells were washed 3 times with cold PBS, and centrifuged to discard the supernatant. TUNEL staining was based on Annexin-V-FITC Apoptosis Detection Kit (556547, Surej Biotechnology Co., Ltd., Shanghai, China). Staining solution was prepared by mixing Annexin-V-FITC, PI, HEPES buffer solution and Annexin-V/PI dye solution at 1:2:50 ratio. Cells at 1 × 10^6^ cells/100 μL were left to stain for 15 min at room temperature and HEPES buffer solution (1 mL) was added and mixed homogeneously. Apoptotic cells were observed by flow cytometry (Bio-Rad ZE5, Hercules, CA, USA). The absorption and excitation wavelengths of FITC were 488 nm and 525 nm, respectively; the absorption and excitation wavelengths of PI-DNA were 535 nm and 615 nm, respectively.

### Statistical analysis

Data were analyzed by SPSS version 21.0 (IBM, Chicago, IL, USA) and were expressed as a mean ± standard deviation. A comparison of data from 2 groups was performed by an unpaired t test, while 3 or more groups were analyzed by one-way analysis of variance (ANOVA) and Tukey's post hoc test. Data comparison between different time points was performed by repeated measures of ANOVA and Bonferroni post hoc test. Differences were considered statistically significant when *p* < 0.05.
